# Tumor monocyte cross talk promotes lymphoma cell resistance to chemotherapy

**DOI:** 10.1186/2051-1426-1-S1-P179

**Published:** 2013-11-07

**Authors:** Zhe (Jenny) Zhang, Peggy A Bulur, Michael P Gustafson, Dennis A Gastineau, Allan B Dietz, Yi Lin

**Affiliations:** 1 Division of Hematology, Department of Medicine, Mayo Clinic, Rochester, MN, USA; 2 Division of Transfusion Medicine, Department of Laboratory Medicine and Pathology, Mayo Clinic, Rochester, MN, USA; 3 Division of Experimental Pathology, Mayo Clinic, Rochester, MN, USA

## Background

Immune editing is a major mechanism used by tumors to promote its survival. We have reported previously the presence of a novel phenotype of immunosuppressive monocytes (CD14+HLA-DR low/neg) in a number of cancers. Increased presence of these cells was associated with decreased treatment response and OS. Here we report an in vitro model to examine tumor monocyte cross talk and demonstrate that these monocytes may directly promote tumor cells’ resistance to chemotherapy.

## Method

Monocytes from healthy donors were co-cultured with lymphoma cell lines (OCI-Ly1, OCI-Ly3, OCI-Ly7, OCI-Ly10, Su-DHL2, Su-DHL6, Jeko-1, Granta-519) with or without doxorubicin (DOX). Cultured cells were assessed for phenotype, viability and proliferation.

## Results

Monocytes converted to CD14+HLA-DRlow/neg phenotype by co-culture with B cell lymphoma lines in 5 of the 8 cell lines tested. B cells from healthy donors did not down regulate HLA-DR expression. The HLA-DR suppression was not due to the known regulatory effects of IL-10 as we found no correlation between the decreased monocyte HLA-DR expression and IL-10 concentration in supernatants. Thus, the magnitude of DR loss is mediated by a yet undetermined mechanism. DOX incubation induced apoptosis, decreased viability and prevented proliferation of all of the cell lines. Co-culture with monocytes improved the lymphoma cell survival in 3 of the 4 lymphoma cell lines (for example, untreated Granta-519 had a 2.1±0.45 fold expansion, that was reduced to 0.39±0.12 when treated with DOX and0.81±0.27 after co-culture with monocytes with DOX. p<0.05, n=11.) Lymphoma cells co-cultured with monocytes had increased arginase I expression. This phenomena was further increased in with DOX and monocyte co-cultures.

## Conclusions

We have demonstrated that certain tumor cells induce phenotype changes in monocytes in an IL-10 independent and a tumor specific way. In those cells where tumors factors mediate monocyte phenotype, the monocytes can promote tumor cell resistance to cytotoxic killing by DOX, thus demonstrating a cross-talk between monocyte and tumor function. We have shown previously that CD14+HLA-DRlow/neg monocytes can suppress lymphocyte function mediated partially through arginase I expression. We found that in an environment of monocyte/tumor cross talk, ARG 1 expression is further increased in a cytotoxic environment. Together, this data demonstrates an active cross talk between monocytes and tumors resulting in multiple mechanisms of tumor resistance to chemotherapy.

